# Hydroxychloroquine in patients with inflammatory and erosive osteoarthritis of the hands: results of the OA-TREAT study—a randomised, double-blind, placebo-controlled, multicentre, investigator-initiated trial

**DOI:** 10.1136/rmdopen-2021-001660

**Published:** 2021-07-02

**Authors:** Claudia Kedor, Jacqueline Detert, Rolf Rau, Siegfried Wassenberg, Joachim Listing, Pascal Klaus, Tanja Braun, Walter Hermann, Stefan Markus Weiner, Frank Buttgereit, Gerd R Burmester

**Affiliations:** 1 Institute of Medical Immunology, Charité Universitätsmedizin Berlin, Berlin, Germany; 2 Rheumatology and Clinical Immunology, Charité Universitätsmedizin Berlin, Berlin, Germany; 3 Rheumatologie, Rheumatologisch-immunologische Praxis, Templin, Germany; 4 Rheumatologie, Rheumazentrum Ratingen, Ratingen, Germany; 5 Epidemiology Unit, Deutsches Rheuma-Forschungszentrum, Berlin, Germany; 6 Rheumatology, Kerckhoff-Klinik GmbH, Bad Nauheim, Hessen, Germany; 7 Medizinische Abteilung, Krankenhaus der Barmherzigen Brüder Trier, Trier, Germany

**Keywords:** hydroxychloroquine, osteoarthritis, analgesics, antirheumatic agents

## Abstract

**Objectives:**

Hand osteoarthritis (OA) is a condition characterised by cartilage degradation and frequently erosive changes. Analgesics and non-steroidal anti-inflammatory drugs are used for symptomatic relief but are often poorly tolerated or contraindicated. Previous publications suggest hydroxychloroquine (HCQ) as a possible treatment for hand OA. The OA-TREAT study aimed to investigate the efficacy and safety of HCQ in patients with inflammatory and erosive hand OA (EOA).

**Methods:**

OA-TREAT was an investigator-initiated, multicentre, randomised, double-blind, placebo (PBO)-controlled trial. Patients with inflammatory and EOA, according to the ACR criteria, with radiographically erosive disease were randomised 1:1 to HCQ 200–400 mg/day or PBO for 52 weeks (W52). Both groups received stable standard therapy. The primary endpoint was Australian Canadian Hand Osteoarthritis Index (AUSCAN) for pain and hand disability at W52.

**Results:**

75 patients were randomised to HCQ and 78 to PBO. At W52, mean AUSCAN pain was 26.7 in HCQ and 26.5 in PBO patients (p=0.92). Hand disability measured by AUSCAN function (mean) was 48.1 in HCQ and 51.3 in PBO patients (p=0.36). Changes in radiographic scores did not differ significantly (p>0.05) between treatment groups. There were 7 serious adverse events in the HCQ and 15 in the PBO group.

**Conclusions:**

OA-TREAT is the first large randomised PBO controlled trial focusing on EOA. HCQ was no more effective than PBO for changes in pain, function and radiographic scores in the 52-week period. Overall safety findings were consistent with the known profile of HCQ.

**Key messages**

**What is already known about this subject?**

- Disease modifying drugs are missing in the treatment of hand osteoarthritis (OA) which is a frequent and often debilitating disease. Hydroxychloroquine (HCQ) has been discussed as an effective treatment.

**What does this study add?**

- OA-TREAT was an investigator-initiated, multicentre, randomised, double-blind, placebo (PBO)-controlled trial focusing exclusively on erosive hand OA (EOA). It showed that hand disability measured by Australian Canadian Hand Osteoarthritis Index (AUSCAN) function and pain measured by AUSCAN pain did not change differently in HCQ compared with PBO patients.
- Changes in radiographic scores did not differ significantly between treatment groups.
- HCQ was generally well tolerated with 7 serious adverse events in the HCQ and 15 in the PBO group.

**How might this impact on clinical practice or further developments?**

- HCQ is not an effective drug in the treatment of EOA. It has a good safety profile in this predominantly elderly population.

## Introduction

Hand osteoarthritis (OA) is a very common disease. The prevalence increases with age.^1 2^ Available treatments are essentially symptomatic, as there are still no disease modifying drugs established for this indication.^3 4^ Common symptoms are pain, stiffness, limited hand function and grip strength resulting in impaired activities of daily living and loss of quality of life. Erosive hand osteoarthritis (EOA) is a subgroup of hand OA with the same but often more severe symptoms characterised by radiographically proven subchondral erosions and cortical destruction, often accompanied by osteophytes, sclerosis, and joint deformity.

The EULAR has published updated recommendations for the management of hand OA.^4^ These recommendations list non-pharmacological measures like education, orthoses and exercises to improve function and muscle strength and pharmacological treatment that aims at pain relief and includes topical non-steroidal anti-inflammatory drugs (NSAIDs), oral analgesics and short courses of NSAIDs. Intra-articular injections of glucocorticoids may be a therapeutic option but should not generally be used. A surgical approach should be considered in advanced or refractory cases. More recently, treatment with 10 mg/day of prednisolone for 6 weeks was shown to be efficacious and safe for the treatment of patients with painful hand OA and signs of inflammation as a short-term treatment for a disease flare-up.^5^ However, in many cases these options are not sufficient or contra-indicated. There is still no disease modifying antirheumatic drug indicated for the treatment of OA, hand OA or hand EOA.

Hydroxychloroquine (HCQ) is a frequently used DMARD (disease-modifying anti-rheumatic drug) therapy in rheumatoid arthritis (RA) and other chronic inflammatory conditions. It plays an important role in the treatment of patients with systemic lupus erythematosus and other connective tissue disorders. In RA, it is used mostly in patients with a milder disease course and as a coadjuvant therapy to other DMARDs like methotrexate.^6^ Originally, it was used in malaria prevention and treatment.

In 1995 a retrospective study with eight patients with EOA refractory to treatment with NSAIDs showed an effect on pain, synovitis and morning stiffness in six of the eight patients.^7^ Other small studies with 7 and 15 patients reported a beneficial effect of HCQ in EOA, whereas another study comparing the use of HCQ and clodronate in patients with EOA could not confirm an effect.^8–10^ In 2018, the HERO (Hydroxychloroquine Effectiveness in Reducing symptoms of Osteoarthritis) Study with 248 patients from the UK showed no significant difference in pain between patients treated with HCQ and placebo (PBO) after 6 months.^11^ Also, in 2018 a randomised, double-blind, PBO-controlled trial with HCQ in 196 patients with symptomatic hand OA in the Netherlands found no effect on hand OA symptoms.^12^ These results were not available at the beginning of our trial. Nevertheless, OA-TREAT provides important confirmatory and new data, since it focused exclusively on hand EOA and included measurement of radiographic progression in this subgroup of patients that is especially at risk for structural damage.

## Methods

### Study design

**Figure 1:**
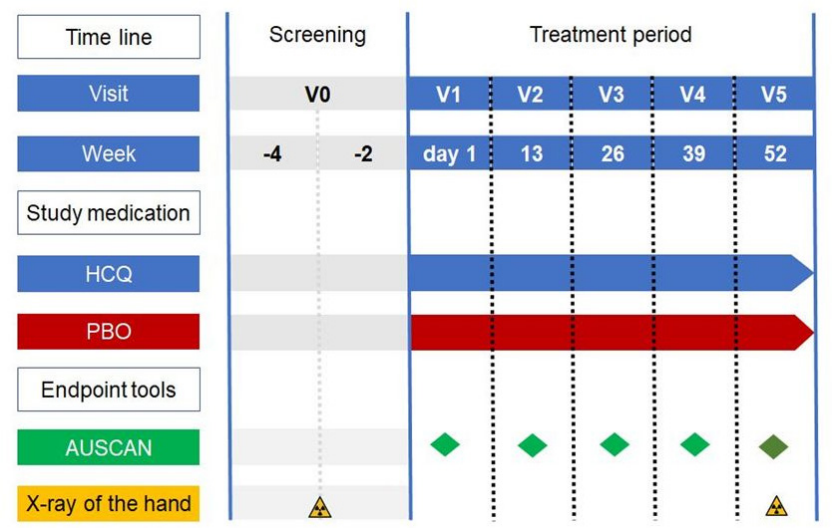
OA-TREAT: study design. AUSCAN, Australian Canadian Hand Osteoarthritis Index; HCQ, hydroxychloroquine; PBO, placebo.

OA-TREAT (acronym for *OA-Treat*ment: a prospective, randomised, double-blind, placebo-controlled clinical trial with hydroxychloroquine in patients with inflammatory osteoarthritis of the hands) was a phase 3b investigator-initiated, multicentre, randomised, double-blind, PBO-controlled trial. The study protocol (previously published),^13^ and its amendments (online supplemental material) were approved by the Berlin (lead site) Ethics Committee (LAGeSo) and by the German Federal Institute for Drugs and Medical Devices (BfArM) and registered on ISRCTN (ISRCTN46445413) and the European Union Drug Regulating Authorities Clinical Trials Database (EudraCT-Number 2011-001689-16). This study was funded by the German Federal Ministry of Education and Research (BMBF). Patients were recruited from November 2013 to January 2017. After randomisation, they were followed up for 52 weeks (intervention period) with additional 4 weeks (safety follow-up period). All participants gave written informed consent before screening. Study design is shown in figure 1.

10.1136/rmdopen-2021-001660.supp1 Supplementary data

### Study centres and participants

The study involved 47 centres in Germany. To be included, patients had to be between 40 and 80 years of age fulfilling the American College of Rheumatology (ACR) criteria for inflammatory hand OA^14^ with radiological signs of EOA in one or more finger joints confirmed by a central assessor on hand radiographs (not older than 6 months) and clinical symptoms of inflammatory

OA defined by pain on pressure and/or active joint swelling and/or redness and/or warmth in more than three finger joints despite taking analgesics and/or NSAIDs for more than 3 months. A pain score ≥4 on a numerical rating scale (0–10) and limitation in function defined as ≥26 using the Australian Canadian Hand Osteoarthritis Index (AUSCAN)-function on a numerical rating scale (0–10) were required. Medication with NSAIDs/cyclo-oxygenase-2-inhibitors should be kept constant 2 weeks prior to study entry.

Radiograph scoring was made based on radiographs of both hands (not older than 6 months at screening, and not more than 2 weeks apart from week 52) in dorsovolar position. Digitalised X-rays of the left hand and, separately, of the right hand were sent to two rheumatologists (SW, RR) who are experienced in reading and scoring of radiographs. Both readers were blinded to the treatment but not to the time order of the images.^14^ The radiographic scores of both readers were averaged per time point and patient, and these averages were used for comparisons between the treatment arms. The inclusion criterion of erosive OA of the hands was checked at the study centre by the same rheumatologists (SW, RR) (see before). Based on the (original) Kallman score, erosions were recorded as a central collapse of the cortex.

Scoring was based on the original Kallman score, the following joints were scored in each hand: the five distal interphalangeal and four proximal interphalangeal joints, the first carpometacarpal (CMC1) joint and the trapezioscaphoid (TS) joint. The CMC1 and TS joints were not scored for cortical collapse, and the TS joint was not scored for osteophytes and lateral deformity. Individual joints were graded for the presence or absence of sclerosis, cysts, lateral deformity, cortical collapse and narrowing of the space (of the CMC1). Osteophytes and narrowing were differentiated into three grades. The Kallman score assesses osteophytes (0–3) and lateral deformity (0–1) in 20 joints, joint space narrowing (0–3), subchondral sclerosis (0–1) and subchondral cysts (0–1), in 22 joints and erosions (0–1) in 18 joints resulting in a possible score range between 0 and 198.^15^

Excluded were patients who were currently treated with HCQ or had received HCQ in the past for OA of the hands and patients who had not tolerated HCQ (eg, skin disease or malaria prophylaxis) or discontinued HCQ due to eye disease (eg, as assessed by an ophthalmologist). Also excluded were patients suffering or having suffered from secondary OA after one of the following diseases (eg, infectious arthritis, acromegaly, ochronosis, haemochromatosis, gout, etc) or inflammatory joint diseases. An inflammatory rheumatic disease was excluded in patients with positive rheumatoid factor, antibodies against cyclic citrullinated peptide (aCCP), antinuclear antibodies (ANA) (usually low titres) or inflammatory markers as these patients did not fulfil criteria for any other inflammatory rheumatic disease except for erosive OA. The decision about the final diagnosis was the responsibility of the investigator. Patients with lymphoma, leukaemia or any malignancy within the past 5 years except for successfully treated basal cell or squamous epithelial carcinomas of the skin were also excluded. Patients with painful syndrome of upper limbs likely to interfere with monitoring of pain as well as patients with an unstable medical condition which would expose the patient to an unacceptable risk were not allowed to participate. A planned surgery, local injections of finger or hand joints with glucocorticoids or other medications within the previous 3 months and the current intake of oral, intra-articular or systemic glucocorticoids (intravenous, intramuscular) were prohibited. Patients with known retinopathy or hypersensitivity to HCQ or to one of the drugs in this study protocol and treatment with digoxin could not be included. Current participation in another clinical trial or experimental treatment was not allowed. Pregnant and breastfeeding women were also excluded.

### Randomisation and intervention

Patients were randomised 1:1 to the treatment groups (either HCQ sulfate (200 mg/day, 200 and 400 mg every other day or 200 mg two times a day according to body weight) or matching PBO) according to a prespecified randomisation list which contained a consecutive number and the corresponding random code A or B. The patients were randomised according to the random code of next consecutive number. The pharmacy was unblinded and knew the random list with the codes A and B, however only their employees knew whether A or B referred to the active drug HCQ. The study drugs subsequently distributed were only labelled with patient numbers.

For patients weighing between 30 and 49 kg, one capsule of 200 mg HCQ or PBO was given. Patients with a body weight between 50 and 64 kg received one capsule with 200 mg HCQ or PBO as a single dose on day 1 and two capsules with 200 mg HCQ or PBO on day 2 in daily alternation. Patients with a body weight of ≥65 kg receive two capsules with 200 mg HCQ or PBO. PBO medication also consisted of capsules with capsule filler DAC NRF (99.5% mannitol, 0.5% high dispersible silicon dioxide) encapsulated in white opaque capsules packaged the same way as study drug (bottle)

### Outcomes

Data were collected at seven time points (screening, baseline, week 13, 26, 39, 52 and at safety follow-up 4 weeks thereafter). Two coprimary endpoints were defined: Australian-Canadian OA Index (AUSCAN, German version) for pain and hand disability at week 52.^16^ Secondary outcomes included: radiographic progression from baseline to week 52 using the Kallman score^15^ and a modified version that was developed during this study, prior to the reading of the radiographs, to better capture erosive changes (for further details see online supplemental material figure S3); patient’s global assessment of disease activity, patient’s assessment of stiffness and physician’s global assessment of disease activity comparison of pain, function, disability, quality of life, based on Health Assessment Questionnaire,^17^ 36-Item Short Form Health Survey (SF-36),^18 19^ Score for Assessment and Quantification of Chronic Rheumatic Affections of the Hands,^20^ from baseline to week 52; assessment and comparison of the inflammatory status using the following parameters: joint pain and joint swelling, night pain, morning stiffness, local erythema/redness, C reactive protein (CRP) and erythrocyte sedimentation rate (ESR)-levels from baseline to week 26 and 52, comparison of the change of consumption of standard medication (NSAIDs, cyclo-oxygenase-2 inhibitors) within the last 7 days before each visit and safety.

### Power calculation

Changes in the AUSCAN scales pain and hand function which correspond the effect sizes of 0.4 for pain and 0.25 for function were considered to be clinically relevant. Based on these assumptions a sample size of n=101 per group is sufficient to achieve a power of 80% for the multiple endpoint test. Originally, it was planned to investigate a second endpoint, the radiological endpoint as a coprimary endpoint. However, this required much larger sample sizes (n=255 per group), which was not achievable due to recruitment difficulties. Therefore, the study protocol was modified, and this endpoint was dropped as primary endpoint.

### Statistical analysis

All patients who received at least one dose of study drug and did not violate important inclusion or exclusion criteria were included in the efficacy analysis and the safety analysis. According to this rule three patients were excluded from the intention-to-treat population: one patient with joint disease due to haemochromatosis (randomised to the HCQ arm), two patients who had been randomised to PBO but received methotrexate and leflunomide (one of both) prior to enrolment. Safety analysis mentions these patients separately.

In the analysis of radiographic outcomes only patients with complete data on Kallman scores at baseline and week 52 were included.

Missing values of primary or secondary outcome parameters were replaced by multiple imputation techniques. The number of imputations was chosen to be 10. Baseline value of the missing parameter and the last valid value of the parameter were used as covariables.

### Analysis of the primary outcome

The multiple endpoint test according to Läuter and O’Brien (SS-sum test) was applied to compare the baseline adjusted coprimary endpoint (AUSCAN pain and hand function scales) at week 52 between the groups.^21^ Separate analyses of the AUSCAN pain and hand function scales were performed as secondary outcome tests in a second step by means of an analysis of covariance (ANCOVA). To achieve statistical significance, a one-sided p value of the SS-sum test <0.025 and a two-sided p value of the corresponding ANCOVA test <0.05 was needed.

### Analysis of secondary outcomes

ANCOVA was used to compare clinical outcome parameters between treatment groups. As covariable, the corresponding baseline value (or where available the mean of screening and baseline value) was included in the ANCOVA models. A non-parametric ANCOVA was used to compare the radiographic endpoints. Probability plots were used to visualise radiographic outcomes.

### Safety analyses

Adverse events (AE) were coded using MedDRA. The current MedDRA version available at the beginning of OA-TREAT (2nd half of 2013) was applied. Serious AE (SAE) terms are reported separately. AEs, SAEs that occurred before the application of the first study medication were not included in the analysis.

## Results

**Figure 2:**
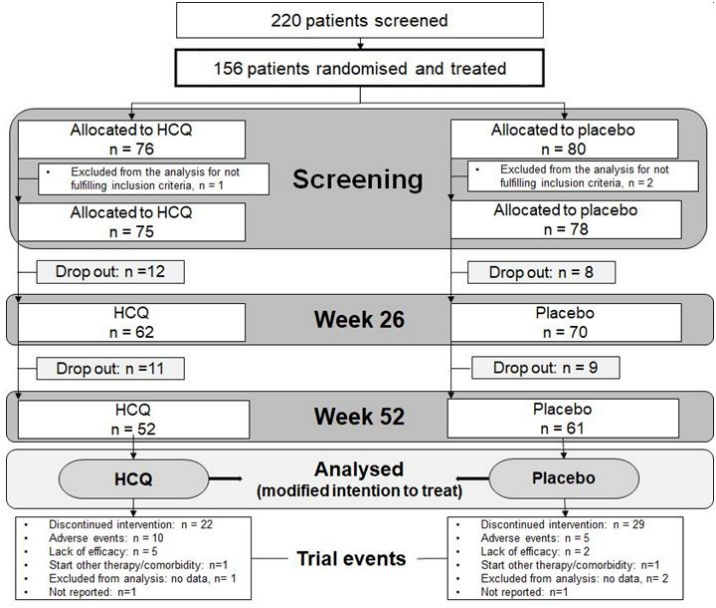
Trial flow. HCQ, hydroxychloroquine.

Of 220 patients screened, 76 were randomised to HCQ and 80 to PBO (figure 2). Distribution of participants per study site are shown in online supplemental figure 1. The other participants were either a screening failure (49 patients did not fulfil inclusion or fulfilled exclusion criteria) and 15 patients decided not to participate in the study (data not collected). Three patients were excluded from the analysis for not fulfilling inclusion criteria. Seventy-five patients were included in HCQ and 78 patients in PBO group. Groups were comparable, except for female gender (90.7% HCQ vs 76.9% PBO). Baseline characteristics are summarised in table 1. Only one participant was unblinded due to generalised rash. At the beginning there were many (minor) protocol violations, significant deviations resulted in three exclusions from analysis. The decision not to include data of these three patients was made before unblinding.

**Table 1:** Baseline characteristics

Parameter	HCQ		PBO	
	n	Value	n	Value
Age in years (mean (SD))	75	52.4 (8.1)	78	50.2 (6.6)
Female gender (n (%))	75	68 (90.7)	78	60 (76.9)
Disease duration in years (mean (SD))	75	9.5 (7.5)	78	10.8 (8.8)
RF IgA positive (n (%))	74	3. (4.1)	77	7.0 (9.1)
RF IgM positive (n (%))	74	10.0 (13.5)	77	8.0 (10.4)
Anti CCP-Ab positive (n (%))	74	3.0 (4.1)	77	3.0 (3.9)
ANA positive (n (%))	74	8.0 (10.8)	77	4.0 (5.2)
CRP in mg/L (mean (SD))	74	4.8 (10.4)	77	3.6 (5.9)
ESR in mm/hour (mean (SD))	73	14.2 (11.4)	74	15.0 (14.7)
AUSCAN pain (mean (SD))	75	31.1 (8.2)	78	30.7 (8.9)
AUSCAN function (mean (SD))	75	58.5 (15.5)	78	57.8 (17.1)
AUSCAN stiffness (mean (SD))	74	6.0 (2.6)	78	5.8 (2.2)
Number of swollen joints from the 30 swollen joint count (mean (SD))	75	3.8 (2.8)	78	4.7 (3.9)
Number of tender joints from the 30 tender joint count (mean (SD))	75	11.5 (6.2)	78	10.4 (6.1)
Sum of periarticular soft tissue oedema (mean (SD))	75	2.7 (3.8)	78	1.9 (2.9)
Patient global (mean (SD))	75	6.3 (1.9)	78	6.1 (2)
Physician global (mean (SD))	75	5.6 (1.5)	78	5.6 (1.4)
SF-SACRAH (mean (SD))	75	5.1 (1.9)	78	4.9 (2.1)
HAQ (mean (SD))	75	0.93 (0.5)	78	0.98 (0.5)
SF-36 standardised physical total scale (mean (SD))	74	35.7 (8.4)	75	35.4 (9.6)
SF-36 standardised mental total scale (mean (SD))	74	50.9 (10.8)	75	50.0 (10.6)
Morning stiffness in minutes (mean (SD))	75	38.3 (37.1)	78	32.8 (33.5)
Nocturnal pain (n (%))	75	42.0 (56.0)	78	44.0 (56.4)
Kallman score (mean SD)	59	42.5 (20.7)	67	43.6 (19.8)
Kallman score (modified) (mean (SD))	59	47.7 (26.0)	67	48.2 (24.2)
Erosion score (modified) (mean (SD))	59	10.0 (9.2)	67	9.8 (8.0)
Original erosion score (mean SD)	59	4.9 (3.1)	67	5.2 (3.1)
Osteophytes (mean (SD))	59	13.1 (7.4)	67	13.4 (7.5)
Joint space narrowing (mean (SD))	59	17.0 (7.7)	67	16.9 (7.2)
Subchondral sclerosis (mean (SD))	59	2.1 (2)	67	2.2 (2.1)
Subchondral cysts (mean (SD))	59	2.9 (2.4)	67	3.7 (2.5)
Lateral deformity (mean (SD))	59	2.5 (2.2)	67	2.2 (2.0)

ANA, antinuclear antibodies; AUSCAN, Australian Canadian Hand Osteoarthritis Index; CCP-Ab, cyclic citrullinated peptide antibodies; CRP, C reactive protein (normal range <5); ESR, erythrocyte sedimentation rate; HAQ, Health Assessment Questionnaire; HCQ, hydroxychloroquine; PBO, placebo; RF, rheumatoid factor; SF-36, 36 Item Short Form Health Survey; SF-SACRAH, Short Form Score for the Assessment and Quantification of Chronic Rheumatoid Affections of the Hands.

The reliability of the radiographic scoring was only moderate. The intraclass correlation coefficients was 0.76 for the Kallman total score.

Even after taking minor mismatches of the AUSCAN scores of pain and function into account and of using a powerful multiple endpoint test no significant difference was found in the outcome of both scales between the treatment arms (table 2, p=0.63).

**Table 2:** Primary outcome week 52

Outcome	Adj. mean HCQ (95% CI)	Adj. mean PBO (95% CI)	P value multiple endpoint	P value separate comparisons	Difference adj. group means (95% CI)
AUSCAN pain	26.7 (23.9 to 29.4)	26.5 (23.9 to 29.1)	0.63	0.92	0.2 (−3.5 to 3.9)
AUSCAN function	48.1 (43.0 to 53.2)	51.3 (46.6 to 56.0)		0.36	−3.2 (−10.0 to 3.6)

By means of analysis of covariance baseline adjusted mean values at week 52, baseline adjusted mean differences and their corresponding 95% CI were calculated.

Adj, adjusted; AUSCAN, Australian Canadian Hand Osteoarthritis Index; HCQ, hydroxychloroquine; PBO, placebo.

AUSCAN score for pain improved in both treatment groups, but there was no statistically significant difference between treatment groups (p=0.92). There was also some improvement in function (AUSCAN score function). However, again after taking minor mismatches at baseline into account and using ANCOVA method the outcome of the AUSCAN score for function did not differ significantly between both treatment arms (p=0.36). Table 2 shows the results of the primary endpoint.

Concerning the secondary endpoints, there was no statistically significant difference between HCQ and PBO, except for ESR (p<0.01) in favour of HCQ and morning stiffness (p=0.001) in favour of PBO at week 52 (table 3). At week 26, there was no meaningful change in any parameter (online supplemental tables S1 and S2).

**Table 3:** Secondary outcome week 52

Outcome	Adj. mean HCQ	95% CI HCQ		Adj. mean PBO	95% CI PBO		P value HCQ vs PBO	Difference adj. group means (95% CI)
AUSCAN stiffness	4.8	4.2	5.4	5.0	4.4	5.5	0.62	−0.2 (−1.0 to 0.6)
Tender joint counts	6.4	4.8	7.9	7.1	5.4	8.7	0.49	−0.7 (−2.6 to 1.3)
Swollen joint counts	2.0	1.3	2.7	2.1	1.4	2.7	0.93	0.0 (−1.0 to 0.9)
Periarticular soft tissue oedema	1.2	0.7	1.6	1.3	0.8	1.9	0.61	−0.2 (−0.8 to 0.5)
**ESR in mm/hour**	**8.2**	**6.9**	**9.6**	**11.7**	**10.1**	**13.5**	**<0.01**	−**3.5 (−6.9 to −1.3**)
CRP in mg/L	2.1	1.6	2.6	2.6	2.1	3.2	0.15	−0.5 (−2.4 to 1.2)
HAQ	0.9	0.8	1.0	0.8	0.7	0.9	0.46	0.1 (−0.1 to 0.2)
Physician global	3.2	2.8	3.6	3.5	3.0	3.9	0.39	−0.3 (−0.9 to 0.3)
Patient global	4.5	3.9	5.1	5.2	4.6	5.8	0.14	−0.6 (−1.4 to 0.2)
SF-SACRAH	4.00	3.5	4.5	4.3	3.8	4.7	0.38	−0.3 (−1.0 to 0.4)
SF-36 mental	48.8	46.6	51.0	50.8	48.7	52.8	0.22	−1.9 (−5.0 to 1.2)
SF-36 physical	39.8	38.0	41.6	39.9	38.2	41.6	0.95	−0.1 (−2.6 to 2.4)
**Morning stiffness in minutes**	**30.2**	**24.0**	**36.3**	**16.3**	**10.3**	**22.3**	**0.001**	**13.9 (5.3 to 22.5**)

By means of analysis of covariance baseline adjusted mean values at week 52, baseline adjusted mean differences and their corresponding 95% CI were calculated.

Adj, adjusted; AUSCAN, Australian Canadian Hand Osteoarthritis Index; CRP, C reactive protein; ESR, erythrocyte sedimentation rate; HAQ, Health Assessment Questionnaire; HCQ, hydroxychloroquine; PBO, placebo; SF-36, 36 Item Short Form Health Survey; SF-SACRAH, Score for Assessment and Quantification of Chronic Rheumatic Affections of the Hands.

**Figure 3:**
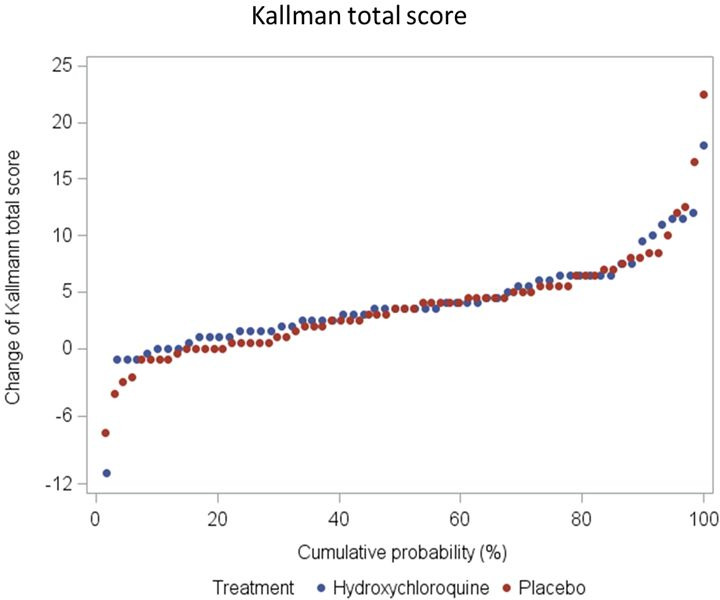
Kallman total score change between baseline and week 52.

**Figure 4:**
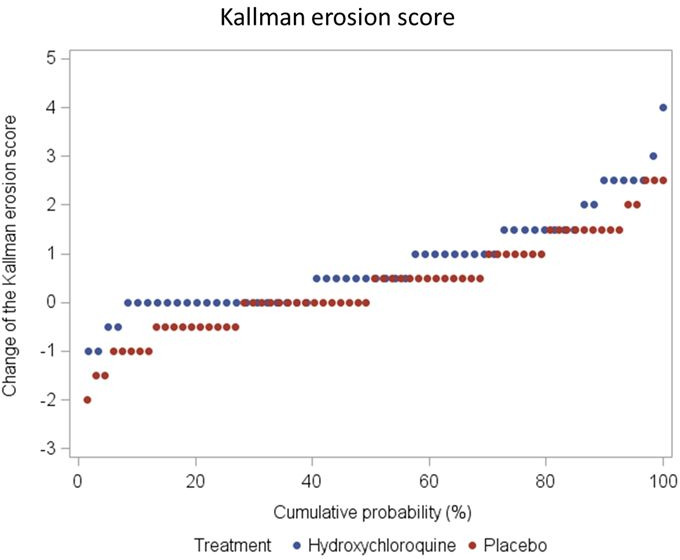
Kallman erosion score change between baseline and week 52.

Also, regarding radiological progression (Kallman total score, erosions, osteophytes, joint space narrowing, lateral deformity, subchondral sclerosis and subchondral cysts) after 52 weeks of treatment there was no statistically significant difference between HCQ and PBO (table 4, figures 3 and 4, online supplemental table S3 and figure S2).

**Table 4:** Radiographic outcome week 52

Outcome	Adj. mean HCQ	95% CI HCQ		Adj. mean PBO	95% CI PBO		P value	Difference adj. group means (95% CI)
Kallman total score	47.1	46.0	48.2	46.8	45.7	47.8	0.71	0.3 (−1.2 to 1.9)
Erosion score	5.8	5.6	6.1	5.4	5.2	5.7	0.02	0.4 (0.1 to 0.8)
Osteophytes	14.7	14.3	15.0	14.7	14.4	15.1	0.56	−0.1 (−0.6 to 0.5)
Joint space narrowing	17.9	17.4	18.3	17.9	17.5	18.3	0.96	0.0 (−0.7 to 0.6)
Lateral deformity	2.6	2.4	2.7	2.6	2.4	2.7	0.95	0.0 (−0.2 to 0.2)
Subchondral cysts	3.7	3.3	4.0	3.8	3.5	4.2	0.25	−0.2 (−0.6 to 0.3)
Sclerosis	2.4	2.1	2.7	2.3	2.1	2.6	0.44	0.1 (−0.3 to 0.5)

The Kallman score assesses osteophytes (0–3) and lateral deformity (0–1) in 20 joints, joint space narrowing (0–3), subchondral sclerosis (0–1) and subchondral cysts (0–1), in 22 joints and erosions (0–1) in 18 joints resulting in a possible score range between 0 and 198. By means of analysis of covariance baseline adjusted mean values at week 52 and their corresponding 95% CI were calculated.

HCQ, hydroxychloroquine; PBO, placebo.

Table 5 summarises the changes in NSAID/cyclo-oxygenase-2 inhibitor consumption. A total of 15 patients had an increase in the equivalent NSAID/cyclo-oxygenase-2 inhibitor dose between baseline and week 52 with 7 individuals in the HCQ versus 8 in the PBO group. Nine patients in total had ≥50% NSAID/cyclo-oxygenase-2 inhibitor increase (HCQ: n=4 vs PBO: n=5). Therefore, it was concluded that the administration of NSAIDs/cyclo-oxygenase-2 inhibitors had no statistical impact on the results of the study. At week 52, 57 patients had a decrease in NSAID/cyclo-oxygenase-2 inhibitor administration, 41 took the same dose, and in 40 patients no data are available because they stopped the study prematurely.

**Table 5:** Patients NSAIDs/cyclo-oxygenase-2 inhibitors administration: decrease and increase between baseline and week 52

NSAID/cyclo-oxygenase-2 inhibitor dose	Total (n)	HCQ (n)	PBO (n)
Increase			
Total	15	7	8
≥50 %	9	4	5
Decrease	57	21	36
Equal	41	24	17
Without data at week 52	40	23	17

*N=number of patients.

HCQ, hydroxychloroquine; NSAID, non-steroidal anti-inflammatory drug; PBO, placebo.

### Safety

Four hundred and eighty-one AE occurred during the study, 245 in the HCQ group and 236 in the PBO group. SAE (n=22) occurred in both groups, although the PBO group presented numerically more events with 15 (68.2 %) individuals compared with the HCQ group: 7 (31.8 %). There were five hospitalisations in the HCQ group (knee operation, peripheral arterial occlusive disease, pneumonia, back pain, nausea), one life-threatening SAE (rash, generalised) and one death, which occurred at week 22 in the HCQ group due to hyperglycaemic coma, which does not correspond to the known opposite influence on the glycaemic profile of HCQ therapy. There were 11 hospitalisations in the PBO group (acute renal failure, OA, peripheral nerve operation, bunion operation, foot operation, positional vertigo, sleep apnoea syndrome, fall, atrial fibrillation, acquired dacryoadenitis, syncope), four SAEs were due to significant risk by investigator’s judgement (age-related macular degeneration, colour blindness, maculopathy, overdose).

Out of the patients described above, five patients had their treatment discontinued (diabetic hyperglycaemic coma, peripheral arterial occlusive disease, generalised rash, syncope), one patient had to be discontinued due to study drug overdose. Six (mentioned before) had their treatment paused (14 days) because of pneumonia, and age-related macular degeneration, and less than 14 days because of acute renal failure, peripheral nerve operation, positional vertigo and atrial fibrillation). One (not mentioned before) had her treatment paused due to nausea (less than 14 days).

## Discussion

OA-TREAT is the first randomised PBO controlled trial to evaluate the effectiveness of HCQ compared with PBO focusing exclusively on severe and refractory inflammatory erosive OA of the hands, defined as persisting symptoms of digital inflammatory OA (pressure pain of the joints and/or florid joint swelling and/or redness and/or warmth) with more than three finger joints for more than 3 months despite taking analgesics and NSAIDs.

There was no difference in both coprimary endpoints AUSCAN pain and AUSCAN function between treatment with HCQ and PBO.

In the randomised, double blind, PBO-controlled Dutch study published in 2018.^12^ Ninety-eight patients in each group with painful hand OA fulfilling ACR criteria for hand OA and confirmed by radiographic evidence of OA at least grade 1 in two joints of the hands according to the Kellgren and Lawrence Classification were treated with HCQ 400 mg/day or PBO over 24 weeks. No superior effect of HCQ was found to PBO in reducing pain as measured on a visual pain scale. Also, no effect could be observed in change of AUSCAN and Arthritis Impact Measurement Scale 2 Short Form after 24 weeks of treatment.

In the HERO study from the UK, also published in 2018, 124 patients in each group were treated with HCQ or PBO over 1 year. The inclusion and exclusion criteria of HERO were comparable to those of the OA-TREAT trial. Both trials required a clinical diagnosis of hand OA confirmed by ACR criteria and radiographic evidence of OA as well as a pain level of >4 on a scale of 0–10. Use of analgetic medication should be kept constant for 4 weeks (HERO) versus 2 weeks (OA-TREAT) before baseline and could be used as needed during both trials. In the HERO Study HCQ dose was adjusted according to the actual ideal body weight of the patient whereas it remained constant based on initial body weight in OA-TREAT. It is worthwhile to mention that some important baseline parameters are very similar in both studies: for example, the (original) baseline Kallman score was 42.5 for HCQ and 43.8 for PBO in OA-TREAT and 42.7 and 43.9 in HERO and mean visual analogue scale pain was 6.3 and 6.1 versus 6.9 and 6.8, whereas others like AUSCAN pain and AUSCAN function were about 2.5 times higher in our study.

Primary outcome criteria in the HERO study were pain and the AUSCAN pain score. In contrast, there were two outcome criteria in OA-TREAT: the AUSCAN score for pain, like in the HERO study, and the AUSCAN score for disability at week 52. But in summary all primary (and secondary) clinical outcome parameters showed no significantly different changes in both studies.

The most important difference between HERO and OA-TREAT was that the latter required the inclusion of at least one erosion. The (original) Kallman score describes erosions as a central collapse of the cortex^15 22^ and assesses them as present or not (1 or 0). A change of the erosion size cannot be recorded. Therefore, it contributes with a maximum score of 18 less than 10% of the overall score of 198 points, although the bone-destructive process is the most impressive change in EOA and the feature most likely to respond to treatment.

At the time this study was designed and approved, there were no published data for randomised controlled trial with HCQ for the treatment of hand OA. Our findings are in line with the two publications quoted above from the Netherlands and from the UK published in 2018, all showing the same result that HCQ is not superior to PBO for the treatment of clinical signs and symptoms of hand OA.

The hallmark of our study was to investigate the efficacy of HCQ in erosive OA of the hands. However, also here no treatment effect could be demonstrated clearly showing that HCQ—although usually well tolerated—is not effective to prevent structural damage in hand OA.

Weakness of this study:

Since OA is a slowly progressing disease, the observed time of 52 weeks for radiological progression may have been too short.

To collect and compare the inflammatory status the following parameters were used: joint pain, joint swelling, nocturnal pain, morning stiffness, local erythema/redness, CRP and ESR levels from baseline to weeks 26 and 52. A systematic ultrasound or MRI might have been a superior assignment of active inflammation, but this was beyond the means of our trial. The inclusion of these techniques would have increased the total cost of the trial far beyond the limited public funding for the trial.

Thus, the search for the underlying mechanism that cause the disease and for disease modifying treatments must continue. Presumably, tackling inflammatory mechanisms appears to be helpful here, not only suggested by the short-term treatment success of glucocorticoids,^5^ but also by the interesting finding that tumour necrosis factor inhibitor treatment is associated with a lower risk of hand OA progression in patients with RA.^23^

A previous study, the digital osteoarthritis in refractory hand OA-study (DORA), evaluating adalimumab in patients with refractory hand OA, failed to demonstrate any clinical 50% improvement after 6 weeks. There were also no statistically significant differences for any of the secondary outcomes (number of painful joints and of swollen joints, morning stiffness, patient global assessments, functional index for hand OA and consumption of analgesics).^24^

## Conclusion

In this study, we could confirm the data of recent publications showing that HCQ is not more effective than PBO for the treatment of (erosive) hand OA and therefore cannot be recommended as a disease modifying anti-OA drug.

## Data Availability

Data are available upon reasonable request. Data are available upon request.
