# Supplementary material for: Hydroxychloroquine in patients with inflammatory and erosive osteoarthritis of the hands: results of the OA-TREAT study—a randomised, double-blind, placebo-controlled, multicentre, investigator-initiated trial
Source: RMD Open. 2021 Jul 2;7(2):e001660. doi: 10.1136/rmdopen-2021-001660 (PMC8256837; doi:10.1136/rmdopen-2021-001660)
Supplement: Supplementary data [file rmdopen-2021-001660supp001.pdf]

Supplemental material

Table S1: Secondary outcomes week 26

Outcome	Adj. mean HCQ	95%-CI HCQ		Adj. mean PBO	95%-CI PBO		p-value HCQ vs. PBO
AUSCAN pain	26.7	24.4	29.0	25.9	23.8	28.1	0.66
AUSCAN function	51.1	47.0	55.1	51.4	47.8	54.9	0.92
AUSCAN stiffness	4.8	4.3	5.4	5.1	4.6	5.6	0.52
Tender joint counts	7.2	6.0	8.4	6.9	5.7	8.2	0.79
Swollen joint counts	2.2	1.7	2.7	1.9	1.4	2.4	0.51
Periarticular soft tissue edema	1.6	1.2	2.0	1.3	0.9	1.7	0.3
ESR in mm/h	9.2	8.0	10.6	10.1	8.8	11.5	0.39
CRP in mg/l	2.3	1.9	2.8	2.4	2.0	2.9	0.79
HAQ	0.89	0.8	1.0	0.9	0.8	1.0	1
Physician global	3.4	3.0	3.7	3.9	3.5	4.2	0.05
Patient global	4.9	4.3	5.4	5.3	4.8	5.9	0.24
SF-SACRAH	4.2	3.8	4.6	4.2	3.8	4.6	0.99
SF36 mental	50.9	48.8	53.0	49.8	47.7	51.8	0.44
SF36 physical	39.3	37.6	41.0	40.0	38.2	41.8	0.56
Morning stiffness in minutes	43.1	26.9	59.3	22.7	7.8	37.5	0.07

Adj. = adjusted; CI = confidential interval; HCQ = hydroxychloroquine; PBO = placebo; vs = versus; AUSCAN = Australian Canadian hand osteoarthritis index; ESR = erythrocyte sedimentation rate; CRP = C-reactive protein; HAQ = health assessment questionnaire; SF-SACRAH = short form score for the assessment and quantification of chronic rheumatoid affections of the hands; SF36 = 36-Item short form health survey.

Table S2: Secondary outcome nocturnal pain

Study time point	HCQ	PBO	p-value HCQ vs. PBO
Week 26	39.9 %	37.7 %	0.85
Week 52	32.7 %	29.9 %	0.79

HCQ = hydroxychloroquine; PBO = placebo; vs. = versus

Table S3: Radiographic outcome week 52

Outcome	Adj. mean HCQ	95%-CI HCQ		Adj. mean PBO	95%-CI PBO		p-value
Kallman score (modified)	53.6	52.1	55.1	52.8	51.4	54.2	0.24
Erosion score (modified)	12.3	11.7	13.0	11.5	10.8	12.1	0.02
Osteophytes	14.7	14.3	15.0	14.7	14.4	15.1	0.56
Joint space narrowing	17.9	17.4	18.3	17.9	17.5	18.3	0.96
Lateral deformity	2.6	2.4	2.7	2.6	2.4	2.7	0.95
Subchondral cysts	3.7	3.3	4.0	3.8	3.5	4.2	0.25
Sclerosis	2.4	2.1	2.7	2.3	2.1	2.6	0.44

The Kallman score assesses osteophytes (0 - 3) and lateral deformity (0 - 1) in 20 joints, joint space narrowing (0 - 3), subchondral sclerosis (0 - 1) and subchondral cysts (0 - 1), in 22 joints and erosions (0 - 1) in 18 joints resulting in a possible score range between 0 and 198. The modified Kallman score was developed during this study to better display the erosions, scoring them on a 0 - 5 scale thus leading to a score range of 0 to 270. Adj. = adjusted; CI = confidential interval; PBO = placebo.

We proposed a modified score that weighs this parameter on a range from zero to five leading to a maximum score of 90 and thereby contributing one third to the total

score between zero and 270. But also using the modified Kallman score, there was no statistical difference between the HCQ and the PBO group. Only the modified Kallman erosion score showed a trend in favour of the placebo group (supplemental material figures S1 and S2).

As the modified Kallman score is not yet validated we cannot make a final conclusion at this time. This will be the subject of further research by our group.

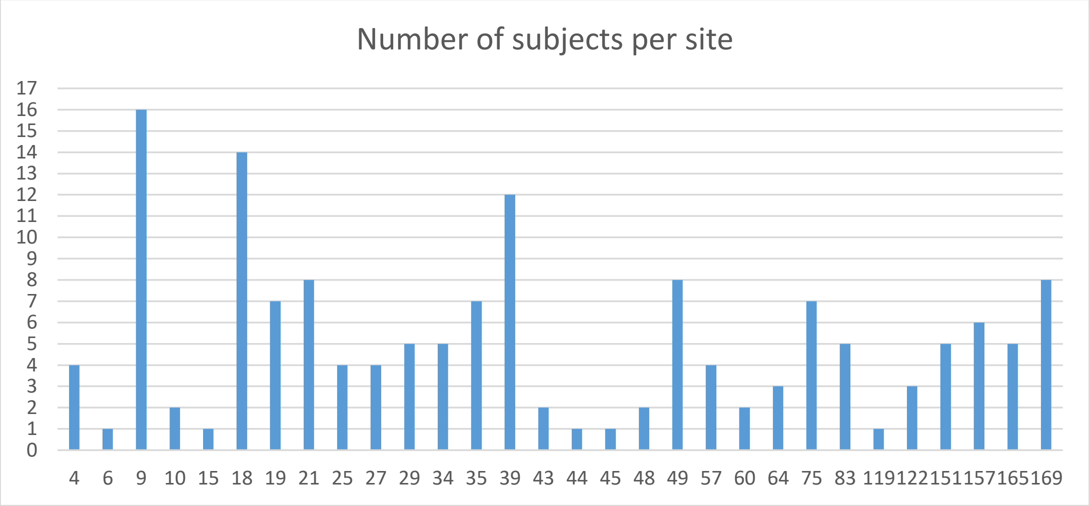

Figure S1: Number of participants included per study centre. From 47 planned centrers, 30 recruited 153 patients. Range from 1 to 16.

The (original) Kallman score describes erosions as a central collapse of the cortex (15, 22) and assesses them as present or not (1 or 0). A change of the erosion size cannot be recorded. Therefore it contributes with a maximum score of 18 less than 10 % to the overall score of 198 points, although the bone-destructive process is the most impressive change in EOA and the feature most likely to respond to treatment. Therefore we proposed a modified score that weighs this parameter on a range from zero to five leading to a maximum score of 90 and thereby contributing one third to the total score between zero and 270. But also with the modified Kallman score there was no statistical difference between HCQ and the PBO group. Only the modified Kallman erosion score showed a trend in favour of the placebo group (supplemental material figures S2 and S3).

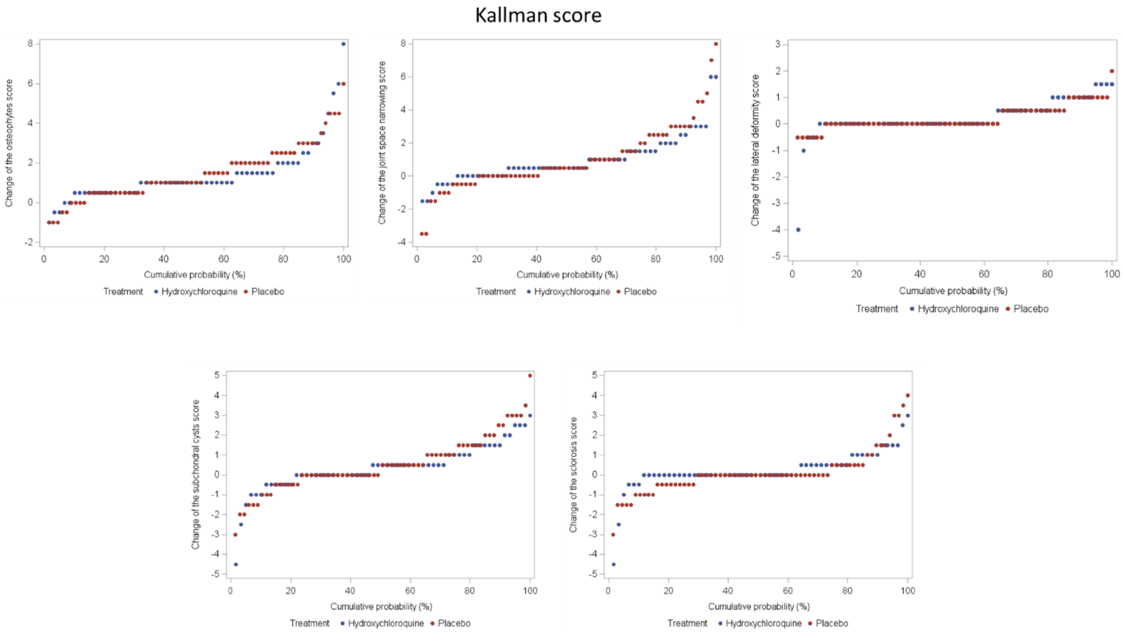

Figure S2: Kallman score: ostophytes, joint space narrowing, lateral deformity, subchondral cysts, subchondral sclerosis

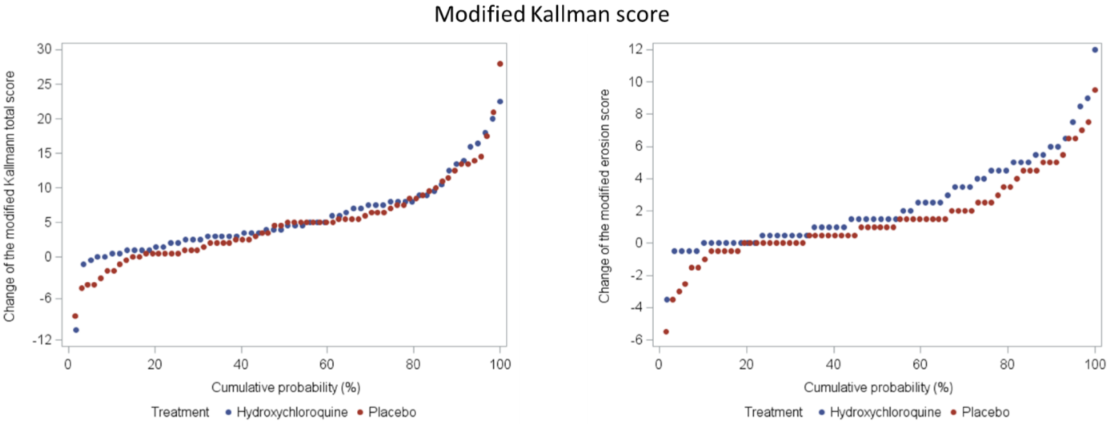

Figure S3: Modified Kallman score*

*The modified Kallman score was developed to assess radiological progression. In contrast to the original Kallman score (15, 16), where erosions are recorded as a central collapse of the cortex and assessed for presence (yes/no) contributing with a maximum of 18 to the overall score of 198 points, in the proposed modified score this parameter became a range from 0 to 5. The intention was to avoid the under-representativeness of bone-destructive processes, believed to be the most likely to be detected after an effective treatment. The modified score´s range was between 0 and 270, and the erosion score contributed to one third of the total score.
